# Taiwanese Older Adults Prefer to Use Antibiotics and Intravenous Infusion at the End of Life based on a Cartoon Version of the Life Support Preferences Questionnaire

**DOI:** 10.3390/ijerph20043430

**Published:** 2023-02-15

**Authors:** Li-Shan Ke, Hui-Chuan Cheng, Chien-Liang Liu, Yu-Chen Ku, Ming-Ju Lee, Yin-Ling Lin, Hsiu-Ying Huang

**Affiliations:** 1 School of Nursing, National Taipei University of Nursing and Health Sciences, Taipei 112, Taiwan; 2 Department of Nursing, Taipei Veterans General Hospital, Taipei 112, Taiwan; 3 Department of Neurology, Taipei City Hospital, Taipei 110, Taiwan

**Keywords:** older adults, life support preferences questionnaire, end-of-life

## Abstract

Asians believe discussing death-related topics is inauspicious and may bring bad luck. It is critical to explore the end-of-life care preferences of the Asian elderly with less-threatening tools. The study examined older adults’ preferences regarding end-of-life treatments by applying a cartoon version of the Life Support Preferences Questionnaire (LSPQ). A cross-sectional survey was conducted to understand older adults’ preferences for end-of-life treatments. A total of 342 older adults participated in the study, comprising 268 elderly patients from a veterans hospital located in northern Taiwan and 74 elderly family members of the patients. Regardless of scenario, cardiopulmonary resuscitation (CPR) had the lowest score, indicating that older adults considered it a less desirable medical treatment. By contrast, antibiotics and intravenous infusions had the highest scores, indicating that older adults tended to prefer them. End-of-life care preferences were significantly different in genders. CPR and surgical preferences of older adults differed significantly with education level. Different demographic characteristics had different end-of-life treatment preferences, and future research may develop advance care planning programs for different attributes. This cartoon version of the LSPQ can help healthcare professionals to understand older adults’ preferences for end-of-life care and warrants further empirical research.

## 1. Introduction

According to the United Nations, the number of older adults aged >65 years was 727 million in 2020, and the global elderly population will double to 1.5 billion by 2050 [1]. The most significant increase is expected to occur in east and southeast Asia, from 261 million in 2019 to 573 million people aged 65 or over in 2050 [2]. Taiwan has one of the largest increases in the proportion of the elderly population [2]. It is necessary to pay attention to the healthcare issues, such as end-of-life care, caused by the rapidly aging population. Experts and scholars believe that when older adults are capable of making decisions, they can express their preferences for end-of-life care, which can improve their dignity, reduce the grief of family members, and minimize the burden on healthcare professionals in clinical care [3,4,5]. However, older adults rarely express their end-of-life care preferences, especially in Asian societies where death-related topics are often considered taboo. Most Asians believe discussing death-related topics is inauspicious and may bring bad luck [6,7]. Therefore, it is critical to explore the end-of-life care preferences of the Asian elderly with less-threatening tools.

The Life Support Preferences Questionnaire (LSPQ) is widely used to examine older adults’ views on end-of-life care. The LSPQ incorporates three concepts—the nature of the disability, potential for recovery, and experience of pain—to form nine clinical scenarios and four medical treatments, resulting in a total of 36 items [8,9]. The readability of the LSPQ was reported to be above the eighth-grade level, and people whose reading skills are below the eighth grade may encounter difficulties in understanding the items of this questionnaire [9]. Studies have noted a few problems when administering the LSPQ to older adults with an average age of 80 years of primary education. For example, these adults had difficulty understanding the items in the questionnaire; the questionnaire’s reliability was also excessively high [10]. A Taiwanese study revealed that the medical treatments listed in the LSPQ, such as antibiotics and cardiopulmonary resuscitation (CPR), were correctly understood by <50% of older adults [11]. The high reliability of the LSPQ recorded in the elderly group implies the possibility of reducing the 36 items of this questionnaire for the elderly population [11]. Additionally, the cultural adaptation of the LSPQ warrants consideration [10].

Pictures can help older adults learn and understand items; nevertheless, Chinese culture regards watching and listening to death-related information as taboo. Cartoon pictures can provide a less threatening approach to learning [12,13]. Scholars have indicated that cartoon pictures can increase readers’ attention and interest through visual language, reduce the burden of reading, and enhance readers’ understanding of the presented content [14]. A study used cartoon pictures to improve older adults’ understanding of end-of-life treatments and reported favorable results [11]. Accordingly, the present study investigated older adults’ preferences regarding end-of-life treatments by applying a cartoon version of the LSPQ.

## 2. Materials and Methods

### 2.1. Design

A cross-sectional survey was used to evaluate older adults’ preferences for end-of-life treatments. The Institutional Review Board of Taipei Veterans General Hospital approved the study (reference number: 2021-02-11C). The study period was from March 2021 to April 2022.

### 2.2. Participants and Settings

Elderly patients hospitalized in the cardiology and hospitalist wards of a veterans hospital in northern Taiwan, and their elderly family members, were recruited to participate in this study. Individuals older than 70 years were included in the study; those with severe vision, hearing, or communication impairment were excluded from the study.

### 2.3. Measurements

**Figure 1:**
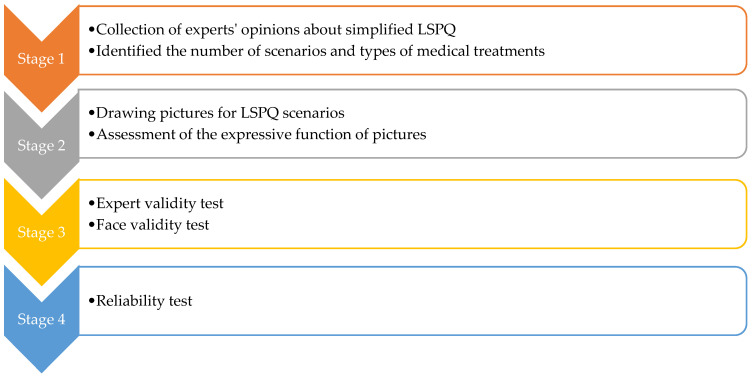
Flowchart of the questionnaire modification process.

The LSPQ used in this study was modified with permission from the original authors. The original LSPQ questionnaire contains nine scenarios: (1) current health status, (2) severe dementia, (3) persistent dyspnea, (4) coma with no chance of recovery, (5) coma with a slight chance of recovery, (6) severe stroke with no chance of recovery, (7) severe stroke with a slight chance of recovery, (8) terminal cancer without pain, and (9) terminal cancer with pain. It also includes four medical treatments: antibiotics, CPR, surgery, and artificial nutrition and hydration [8,9]. The items are scored on a 5-point Likert scale ranging from 1 (“definitely don’t want”) to 5 (“definitely want”) [8,9]. A higher score indicates a higher likelihood that the participant will use the end-of-life treatments. The study used a modified Delphi method to collect the opinions of 11 experts in the fields of palliative medicine, palliative care, geriatric medicine, and geriatric nursing [15]. The questionnaire modification process involved four stages (for the flowchart of the questionnaire modification process, please see Figure 1).

**Figure 2:**
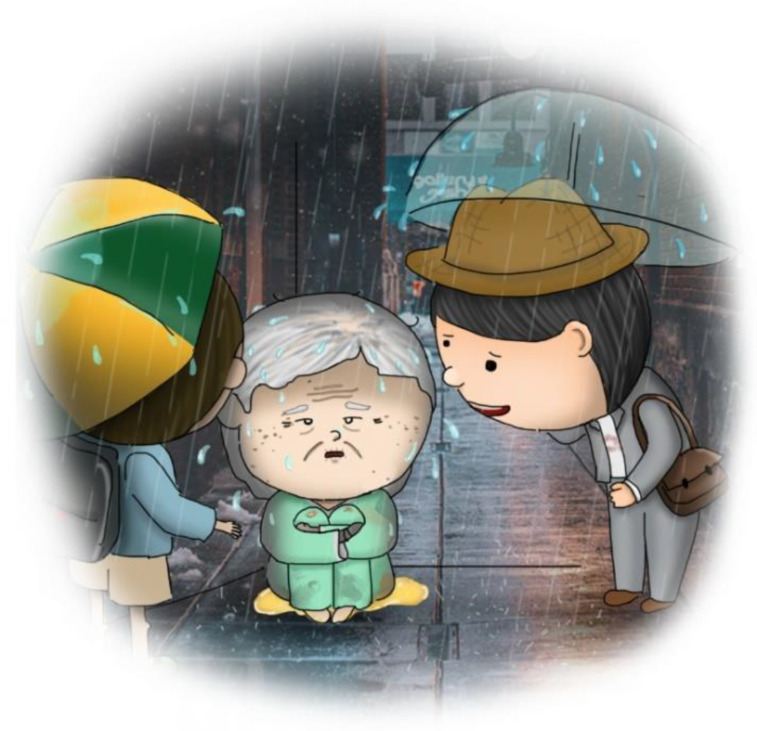
Severe dementia.

**Figure 3:**
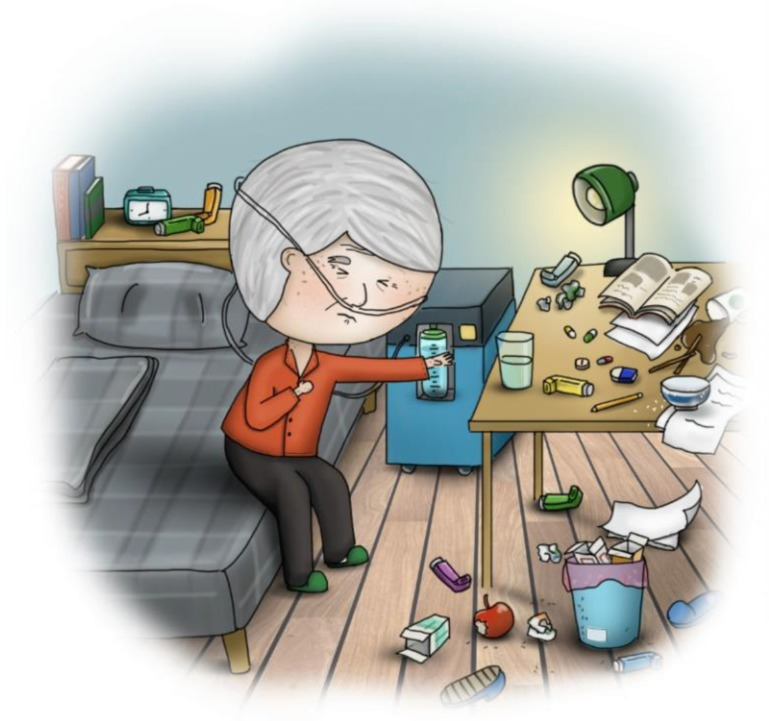
Persistent dyspnea.

**Figure 4:**
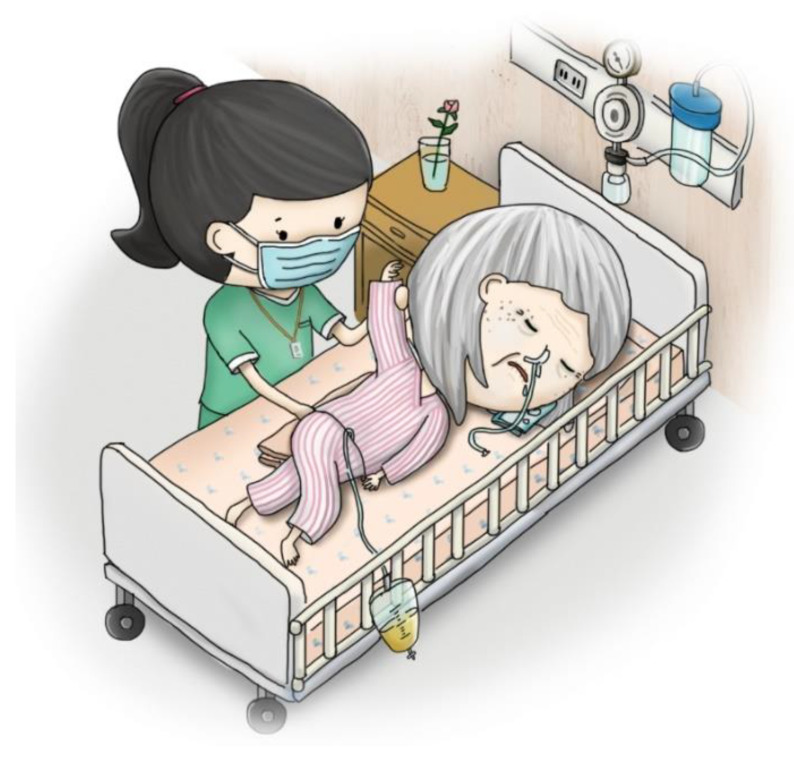
Coma with a slight chance of recovery.

**Figure 5:**
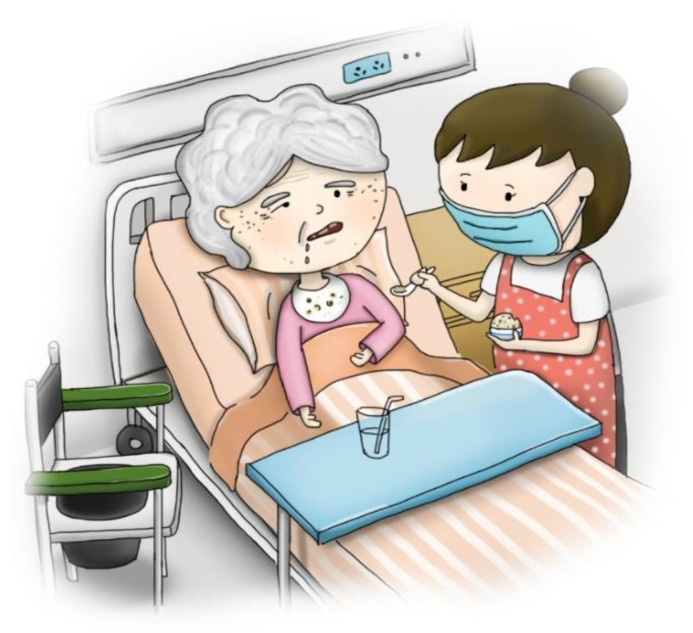
Severe stroke with a slight chance of recovery.

**Figure 6:**
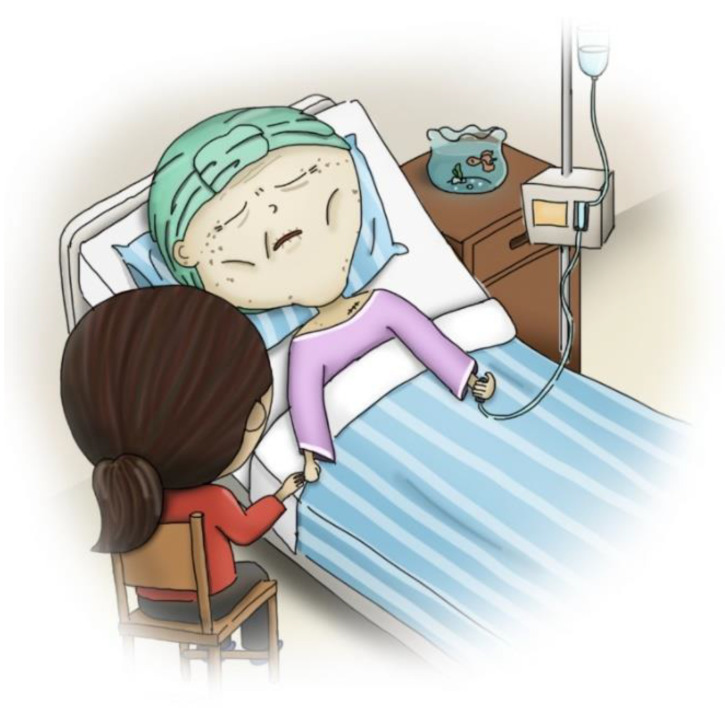
Terminal cancer with pain.

1. Stage one: This stage involved mailing a structured questionnaire to the experts, explaining the research purpose, and asking them to respond with their opinions on reducing the LSPQ items. Experts agreed to remove only similar scenarios; for example, stroke comprises two scenarios: “no chance of recovery” and “slight chance of recovery”. These experts believed that one of the scenarios could be deleted because “no chance of recovery” or “slight chance of recovery” might not make any difference in very elderly adults. Similarly, they recommended deleting one of the two scenarios related to terminal cancer. Consequently, three scenarios were removed: coma with no chance of recovery, severe stroke with no chance of recovery, and terminal cancer without pain. Regarding medical treatments, the experts recommended splitting artificial nutrition and hydration into two separate items, namely intravenous infusion and nasogastric tube feeding, because of the cultural context of Taiwan. Finally, the first draft of the questionnaire, with six scenarios and five medical treatments, was developed, comprising a total of 30 items.
2. Stage two: The six scenarios and five medical treatments were depicted in pictures. The authors repeatedly corresponded with the cartographer regarding the scenarios to be illustrated. After several revisions, five scenario pictures (severe dementia, persistent dyspnea, coma with a slight chance of recovery, severe stroke with a slight chance of recovery, and terminal cancer with pain) were drawn (Figure 2, Figure 3, Figure 4, Figure 5 and Figure 6). Before using these five pictures, the authors invited seven community-dwelling older adults to describe the images they saw. Most of the older adults described Figure 2 as a demented older man who could not find his way home, and Figure 3 as a person who had difficulty breathing and could not get a water cup when he wanted to drink water. Older adults described Figure 4 as a person who was unconscious and could not move by herself, and Figure 5 as an older woman after a stroke who needed help from others to eat and go to the toilet. Regarding Figure 6, most older adults described the person as being very sick and might not survive. The results indicated that these pictures could articulate the five scenarios in LSPQ. For medical treatments, four pictures were adopted to represent antibiotics, CPR, surgery, intravenous infusion, and nasogastric tube feeding [11]. These cartoon pictures and 30 items of the questionnaire constituted the prototype of the questionnaire.

3. Stage three: Experts were invited to comment on aspects of the draft questionnaire, such as picture colors and text descriptions, and were also required to determine expert validity for the draft questionnaire. The experts used the content validity index (CVI) to assess content validity. The CVI incorporates content suitability and text clarity [16]. The CVI value for content suitability was determined to be 1 for all scenarios. Regarding text clarity, the CVI value was determined to be 0.74 for current health status, 0.77 for severe dementia, 0.77 for persistent dyspnea, 0.85 for coma with a slight chance of recovery, 0.74 for severe stroke with a slight chance of recovery, and 0.74 for terminal cancer with pain. The original LSPQ focuses on the willingness of older adults to undergo gallbladder surgery for gallbladder inflammation [8]. However, during the modification process, the experts mentioned that several older adults had undergone cholecystectomy, and thus suggested that the question for surgery be slightly adjusted to the following: “If you had an inflammation of the gallbladder or another organ, would you be willing to undergo surgery?” Regarding the layout and binding part of the questionnaire, each scenario appeared on the left-hand side of the reader, and the medical treatment appeared on the right-hand side so that the reader could know which clinical scenario they answered. Next, an A4 size thick white non-reflective paper was chosen for the printing. Before recruiting participants, seven older adults from the hospital’s cardiology and hospitalist wards with an education level below elementary school were asked to read a cartoon version of the LSPQ. Older adults said cartoon pictures could increase their understanding of the questionnaire questions.
4. Stage four: The reliability of the questionnaire was tested by taking older adults over 70 years old as samples. The Cronbach’s alpha coefficient of each section of the questionnaire was as follows: current health status, 0.73; severe dementia, 0.87; persistent dyspnea, 0.84; coma with a slight chance of recovery, 0.90; severe stroke with a slight chance of recovery, 0.91; and terminal cancer with pain, 0.91. The overall Cronbach’s alpha of the questionnaire was 0.97. The results show that the questionnaire has good reliability.

### 2.4. Data Collection

The researchers explained the purpose of the study to the participants. After the adults had agreed to participate and had signed the consent form, they were asked to complete the questionnaire. If older adults needed semantic assistance when filling out the questionnaire, the researchers would explain it based on the pictures. The questionnaire completion time was approximately 15 min. The location selected for data collection was a single-patient room or a conference room to maintain the participants’ privacy.

### 2.5. Data Analysis

IBM SPSS Statistics for Windows, version 20.0 (IBM Corp., Armonk, NY, USA) was used for data analysis, and statistical significance was set at *p* < 0.05. Descriptive statistics, namely mean, standard deviation, percentage, and rank, were used to characterize each variable. The scores of the same medical treatment in the questionnaire were added to form the scores of five medical treatments, namely antibiotics, CPR, surgery, nasogastric tube feeding, and intravenous infusion (range 6–30 points). In addition, all scores were added together to form the LSPQ total score (range 30–150 points). Univariate analysis of demographic data, LSPQ total score, and subscales were performed using chi-square test, independent *t*-tests or ANOVA, where appropriate. The study used G power 3.1.9.4 software and ANOVA, and fixed effect, special, main effect, and interactions to calculate the sample size. A total of 269 samples were required. When the effect size was at 0.25, the alpha was at 0.05, the power was 0.8, and the number of groups was 4. However, a sample size of 300 is recommended for psychology and psychiatry investigations [17].

## 3. Results

Three hundred forty-three questionnaires were delivered to the eligible participants, and 342 were validly completed, with a response rate of 99.7%. A total of 342 older adults participated in the study, comprising 268 elderly patients and 74 elderly family members. Their average age was 80.13 (7.63) years. Of the participants, 213 were men (62.3%), 129 were women (37.7%), 65.9% were married, and 23.4% were widowed, with most living with their family members. Elementary school and below (44.8%) constituted the largest educational group, and the most predominant religious beliefs were Buddhism (44.0%) and Taoism (23.4%, Table 1).

The results also revealed that across three scenarios (current health status, severe dementia, and persistent dyspnea), the participants’ preferences for the five medical treatments were similar and could be ordered as follows (in ascending order): CPR, nasogastric tube feeding, surgery, intravenous infusion, and antibiotics. Regarding coma with a slight chance of recovery and severe stroke with a slight chance of recovery, the participants ranked the medical treatments in the following order: CPR, nasogastric tube feeding, surgery, antibiotics, and intravenous infusion. Regarding terminal cancer with pain, the treatments were ranked in the following order: CPR, surgery, nasogastric tube feeding, antibiotics, and intravenous infusion. Regardless of scenario, CPR had the lowest score, indicating that the participants considered CPR to be less desirable than the other treatments. The second least desirable medical treatment by the participants was nasogastric tube feeding. By contrast, antibiotics and intravenous injections had the highest scores, indicating that older adults tended to prefer them (Table 2).

Table 1 shows that gender had significant differences in LSPQ total score (*t* = −3.301, *p* ≤ 0.001), scores of antibiotics (*t* = −2.225, *p* < 0.05), CPR (*t* = −3.884, *p* < 0.001), surgery (*t* = −3.081, *p* < 0.01), intravenous infusion (*t* =−2.022, *p* < 0.05), and nasogastric tube feeding (*t* = −3.309, *p* < 0.01). Participants’ LSPQ total score (*t* = −2.205, *p* < 0.05), antibiotics (*t* = −2.197, *p* < 0.05), CPR (*t* = −2.640, *p* < 0.01), and intravenous infusion (*t* = −2.339, *p* < 0.05) scores showed significant differences in age. Additionally, participants’ education levels also differed significantly in CPR (*F* = 6.550, *p* < 0.01) and surgery (*F* = 4.114, *p* < 0.05) scores. Table 3 shows differences in the ratio of males to females in age stratification (*X*^2^ = 13.366, *p* < 0.001) and educational level (*X*^2^ = 25.586, *p* < 0.001).

## 4. Discussion

### 4.1. Discussion

The participants in the present study rated CPR as the least desirable medical treatment, a finding consistent with those of several other studies [10,18,19,20]. With the promotion of palliative care in Taiwan, most older adults no longer want to receive ineffective medical treatments such as CPR, and they value their hospice rights. A study analyzing Taiwan’s health insurance database reported a significant drop in the proportion of older adults with dementia receiving CPR, from 31% in 2010 to 15% in 2013 [21]. A mixed study noted considerable agreement between an elderly Taiwanese population and their surrogates regarding their refusal to undergo CPR in end-of-life care [18]. The end-of-life care of older adults does not require CPR, a fact that has gained the attention of most Taiwanese. By contrast, older adults in the present study were more inclined to use antibiotics, similar to the results of a study in Singapore [19]. An integrative study revealed that older age, a lower education level, and rural living increased the demand for inappropriate antibiotic use among Chinese older adults [22]. Among end-of-life treatments, antibiotics are the least invasive; moreover, the common misconception that antibiotics have preventive and protective effects, or that antibiotics are anti-inflammatory drugs, makes older adults tend to use them [22,23].

In traditional Chinese culture, “feeding before hitting the road” is considered the most basic care for people who will die soon. For example, death-row prisoners typically have a full meal before execution, and starvation or dehydration is believed to be cruel punishment [24]. Chinese culture is deeply embedded in Taiwanese society. In general, Taiwanese patients and their families tend to use artificial nutrition and hydration at the end of life; however, Taiwanese older adults prefer intravenous infusion, and would rather not be force-fed through a nasogastric tube [10]. A study conducted in Singapore also reported that older adults had two different views on artificial nutrition and hydration: up to 74% of hospitalized older adults tended to use intravenous infusions, and only 35% were willing to accept tube feeding [19]. An epidemiological survey of inpatients at 156 public hospitals in 30 provinces in China revealed that 93.1% of the patients received intravenous infusions in 2016 [25]. These results indicate that patients with a Chinese cultural background seem to prefer the use of intravenous infusions. Scholars have observed that Chinese patients’ preference for intravenous infusions may be related to physicians’ lack of knowledge about intravenous infusions or the revenue benefit derived by medical institutions from this practice [26]. Nevertheless, culture is the most dominant factor contributing to people’s misconceptions about intravenous infusions [26]. Chinese people believe that an intravenous infusion can quickly restore physical strength and supplement nutrition [27]. Thus, it is common for patients or their families to request intravenous infusions in Chinese hospitals [28].

The results also reveal that older adults were more unwilling to receive nasogastric tube feeding than to receive intravenous infusions. A possible reason for this unwillingness is that changes in physical appearance sensitize older adults to interpersonal problems, or that threats to body integrity make older adults worry that the nasogastric tube cannot be removed, or that tube placement requires drilling a hole in the body. Chinese culture attaches great importance to interpersonal interaction and body integrity [29], which may cause older adults to prefer intravenous infusions and dislike nasogastric tube feeding.

Gender differs significantly in end-of-life treatment preferences, and men seem to favor end-of-life treatments, similar to previous studies [30,31,32,33]. A study in Switzerland showed that women tend to avoid over-treatment compared with men, and pay more attention to end-of-life planning [31]. The study also noted that the most significant difference between men and women in end-of-life care preferences lies in psychosocial aspects [31]. Women are more likely to use emotional and social support to cope with stress [31]. A recent scoping review showed that women are less likely to like invasive treatments than men, and are more worried about burdening their families, so they tend not to make decisions, while men tend to take control of their own decisions [33]. In addition, the life expectancy of women is longer than men’s, women may have more experience in facing relatives’ end-of-life care, and so they tend to use fewer end-of-life treatments than men [31]. Scholars believe that gender differences may be related to gender roles and expectations dictated by social culture [34]. The study showed that the end-of-life treatment preferences of older adults differed significantly with age. People over 80 years old tend to use end-of-life treatments more than those under 79. This result differs from many studies, which mentioned that the older the elderly, the more inclined not to use life-sustaining measures [35,36]. Our research shows that women comprised 27.5% of participants over 80 and 46.7% of those under 79. It is speculated that older adults in the study had different preferences for end-of-life treatments at different ages, which may come from gender differences.

In this study, the CPR and surgery scores significantly differed with the education level of older adults. Those with a high education level preferred CPR and surgery compared to those with a middle or low level of education. This is similar to the results of the Korean study [20], which showed that older adults with high education tended to use CPR and hemodialysis. This phenomenon is inconsistent with studies in Western countries [31,37]. Studies showed that people with higher education levels tend to have less invasive treatments [31,37]. However, education level does not explain individuals’ complex preferences for end-of-life treatments. Notably, older adults’ decision-making situations or regional cultures must be considered. It is interesting to analyze why people with high education levels prefer CPR and surgery compared to those with low and medium education levels. One possible reason is that people with low education levels have a limited understanding of end-of-life treatments. Relatively, they have fewer requirements for treatments. Another possible reason is that people with a low level of education may have different life experiences that make them pay more attention to natural or cultural beliefs. Undoubtedly, the study’s ratio of men to women also contributed to this result. However, the current consensus is that older adults with high knowledge of advance care planning are more likely to discuss their end-of-life care [38,39]. Thus, improving the understanding of advance care planning among older adults should be the focus of efforts.

### 4.2. Limitations

This study has two limitations. First, although the study recruitment process was to obtain the oral consent of the participants and sign the written consent form before providing the questionnaire, the exceptionally high response rate may create selection bias. Second, the sample of this study came from a veterans hospital in northern Taiwan, and the ratio of males to females in the sample was different from that of the general population, so care must be taken when using the results of this study.

## 5. Conclusions

Older adults are more likely to use antibiotics and intravenous infusions and not CPR or nasogastric tube feeding. Additionally, different demographic groups have different end-of-life treatment preferences, and future research can develop advance care planning programs for different groups. This study highlights the cultural context behind Taiwanese older adults’ perceptions of artificial nutrition and hydration.

## 6. Practice Implications

According to our review of the literature, this is the first study to use cartoon pictures to assist LSPQ in understanding end-of-life care preferences in older adults. In the study, the cartoon pictures also standardized the researchers’ interpretation of the medical treatments. In addition, certain sensitive issues were easily accepted and inherent conflicts easily resolved through cartoon depictions [14]. In end-of-life care, cartoon pictures are suitable for conveying sensitive information and enhancing understanding. The use of the cartoon version of the LSPQ for the elderly population is a relatively new field and warrants further empirical research.

## Figures and Tables

**Table 1:** Univariate analysis of the demographic characteristics of older adults and the LSPQ (N = 342).

Variables	n (%)	Total Score of LSPQ M (SD)	Antibiotics M (SD)	CPR M (SD)	Surgery M (SD)	IV M (SD)	NG Feeding M (SD)
Participant sources		*t* = 0.902	*t* = 0.076	*t* = −0.542	*t* = 1.529	*t* = −0.075	*t* = 1.674
Elderly patients	268 (78.4)	86.42 (25.52)	20.37 (5.32)	13.75 (6.85)	16.99 (5.89)	20.02 (6.02)	15.48 (6.65)
Elderly family members	74 (21.6)	89.63 (29.41)	20.42 (5.81)	13.25 (6.90)	18.21 (6.30)	19.96 (6.40)	17.00 (7.36)
Gender		*t* = −3.301 ***	*t* = −2.225 *	*t* = −3.884 ***	*t* = −3.081 **	*t* = −2.022 *	*t* = −3.309 **
Female	129 (37.7)	81.07 (25.48)	19.55 (5.67)	11.91 (5.87)	15.98 (5.98)	19.15 (6.27)	14.24 (6.86)
Male	213 (62.3)	90.79 (26.28)	20.89 (5.20)	14.71 (7.19)	18.03 (5.88)	20.53 (5.93)	16.75 (6.64)
Age		*t* = −2.025 *	*t* = −2.197 *	*t* = −2.640 **	*t* = −0.930	*t* = −2.339 *	*t* = −0.699
70–79	182 (53.2)	84.32 (25.91)	19.77 (5.46)	12.71 (6.27)	16.97 (5.70)	19.28 (6.25)	15.55 (6.94)
≥80	160 (46.8)	90.17 (26.61)	21.06 (5.29)	14.69 (7.32)	17.58 (6.31)	20.83 (5.82)	16.08 (6.70)
Education level		F = 2.108	F = 0.094	F = 6.550 **	F = 4.114 *	F = 0.108	F = 2.555
Elementary school and below	151 (44.8)	86.00 (25.12)	20.21 (5.32)	13.09 (6.61)	16.99 (5.70)	20.15 (6.23)	15.50 (6.86)
Secondary school	103 (30.6)	84.23 (23.95)	20.51 (5.23)	12.43 (6.21)	16.31 (5.64)	19.85 (5.59)	15.00 (6.38)
Higher education	83 (24.6)	91.99 (30.84)	20.41 (5.92)	15.85 (7.53)	18.80 (6.78)	19.83 (6.55)	17.21 (7.29)
Marital status		F = 1.262	F = 1.073	F = 1.538	F = 2.177	F = 1.196	F = 0.959
Single	18 (5.3)	93.71 (24.46)	22.29 (5.98)	14.41 (7.66)	17.53 (5.71)	22.71 (4.38)	16.76 (7.27)
Married/relationship	222 (65.9)	88.17 (26.62)	20.34 (5.22)	14.06 (7.03)	17.78 (5.98)	19.83 (6.04)	16.06 (6.82)
Divorced	18 (5.3)	85.11 (31.34)	21.00 (7.36)	11.89 (6.17)	16.39 (5.91)	19.67 (7.81)	16.17 (8.73)
Widowed	79 (23.4)	82.62 (24.86)	19.81 (5.38)	12.44 (6.21)	15.84 (6.10)	19.87 (6.14)	14.66 (6.42)
Living condition		F = 0.029	F = 0.965	F = 0.524	F = 0.373	F = 0.481	F = 1.204
Living with family	278 (83.0)	86.93 (27.89)	20.17 (5.45)	13.66 (6.99)	17.32 (6.19)	19.84 (6.24)	15.88 (6.97)
Living in long-term care facility	28 (8.4)	85.62 (19.20)	20.73 (4.78)	12.23 (4.88)	16.31 (4.58)	20.15 (4.51)	16.19 (6.21)
Living alone	29 (8.7)	86.76 (23.93)	21.59 (5.80)	13.41 (7.00)	16.90 (5.60)	21.00 (6.26)	13.86 (6.20)
Religion		F = 0.142	F = 0.148	F = 0.579	F = 0.328	F = 0.409	F = 0.469
None	67 (20.1)	88.12 (27.83)	20.42 (5.62)	14.49 (7.18)	17.05 (6.59)	19.68 (6.05)	15.98 (6.50)
Buddhism	147 (44.0)	86.09 (25.48)	20.41 (5.38)	13.55 (6.86)	17.10 (5.68)	19.71 (5.87)	15.31 (6.65)
Taoism	78 (23.4)	86.89 (26.48)	20.00 (5.03)	12.97 (6.73)	17.21 (5.72)	20.59 (6.50)	16.36 (7.48)
Christian/Catholic	42 (12.6)	88.53 (28.46)	20.63 (6.30)	13.51 (6.78)	17.95 (6.79)	20.22 (6.54)	16.21 (7.20)

* *p* < 0.05, ** *p* < 0.01, *** *p* < 0.001.

**Table 2:** Older adults’ preferences about end-of-life treatments (N = 342).

Variable (Range 1–5 Points)	Mean (SD)	Ranking
Currently healthy		
Antibiotics	4.20 (0.87)	5
Cardiopulmonary resuscitation	2.62 (1.43)	1
Surgery	3.65 (1.11)	3
Intravenous infusion	3.99 (1.02)	4
Nasogastric tube feeding	2.99 (1.34)	2
Severe dementia		
Antibiotics	3.58 (1.18)	5
Cardiopulmonary resuscitation	2.38 (1.37)	1
Surgery	3.03 (1.27)	3
Intravenous infusion	3.47 (1.25)	4
Nasogastric tube feeding	2.72 (1.30)	2
Persistent dyspnea		
Antibiotics	3.77 (1.03)	5
Cardiopulmonary resuscitation	2.39 (1.32)	1
Surgery	3.05 (1.17)	3
Intravenous infusion	3.55 (1.13)	4
Nasogastric tube feeding	2.79 (1.29)	2
Coma with a slight chance of recovery		
Antibiotics	2.99 (1.35)	4
Cardiopulmonary resuscitation	2.16 (1.26)	1
Surgery	2.60 (1.29)	3
Intravenous infusion	3.06 (1.35)	5
Nasogastric tube feeding	2.48 (1.30)	2
Severe stroke with a slight chance of recovery		
Antibiotics	3.06 (1.33)	4
Cardiopulmonary resuscitation	2.16 (1.22)	1
Surgery	2.64 (1.24)	3
Intravenous infusion	3.07 (1.31)	5
Nasogastric tube feeding	2.51 (1.28)	2
Terminal cancer with pain		
Antibiotics	2.78 (1.38)	4
Cardiopulmonary resuscitation	1.98 (1.19)	1
Surgery	2.33 (1.25)	2
Intravenous infusion	2.87 (1.38)	5
Nasogastric tube feeding	2.33 (1.27)	3

**Table 3:** Differences in male-to-female ratios by age stratification and educational level (N = 342).

	Gender		*X* ^2^	*p*
	Female n (%)	Male n (%)		
Age			13.366	<0.001
70–79	85 (46.7)	97 (53.3)		
≥80	44 (27.5)	116 (72.5)		
Education level			25.586	<0.001
Elementary school and below	77 (51.0)	74 (49.0)		
Secondary school	35 (34.0)	68 (66.0)		
Higher education	15 (18.1)	68 (81.9)		

## Data Availability

The data presented in this study are available on request from the corresponding author. The data are not publicly available due to ethical issues.
